# ﻿Not so deep in the rainforest: two new species of *Anastrepha* (Diptera, Tephritidae) and a pictorial key to species from Amazonas state, Brazil

**DOI:** 10.3897/zookeys.1244.150382

**Published:** 2025-07-10

**Authors:** Keiko Uramoto, Alexandre S. Araújo, Francisco C. Costa-Silva, Neliton M. Silva, Marcoandre Savaris, Roberto A. Zucchi

**Affiliations:** 1 Escola Superior de Agricultura “Luiz de Queiroz”, Universidade de São Paulo, Piracicaba, SP, Brazil Universidade de São Paulo Piracicaba Brazil; 2 Faculdade de Ciências Agrárias, Universidade Federal do Amazonas, Manaus, AM, Brazil Universidade Federal do Amazonas Manaus Brazil

**Keywords:** Brazilian Amazon, diversity, fruit flies, geographical distribution, taxonomy

## Abstract

Two new species, *Anastrephadorsidentata* Uramoto, Zucchi, Araújo & Savaris and *A.norrbomi* Uramoto, Zucchi, Araújo & Savaris, from a fragment of the Amazon Rainforest in the city of Manaus, state of Amazonas, Brazil, are described and illustrated. Both species were collected in McPhail-type traps hanging from trees on the campus of Federal University of Amazon. A pictorial key to 47 species of *Anastrepha* recorded in Amazonas state is provided.

## ﻿Introduction

Amazon forest, as legally defined, encompasses approximately 5 million km^2^ of the territory of Brazil, comprising 59% of the country and including part or all of nine states. Of these states, Amazonas is the largest (about 1.5 million km^2^) and borders on five other states (Acre, Mato Grosso, Pará, Rondônia, and Roraima) and three countries (Colombia, Peru and Venezuela).

In general, records of *Anastrepha* species in the state of Amazonas have been sporadic or restricted to the vicinity of urban centers (see Silva et al. 2023). This limitation is primarily due to the difficulty of accessing the diverse ecosystems of the Amazon. The characteristics of the region and the state’s vast territorial expanse pose challenges for collecting, which impede our understanding of the local diversity of species.

A more comprehensive understanding of diversity requires a coordinated effort to collect specimens across the diverse ecosystems that comprise the Amazon biome. This effort involves the implementation of continuous surveys, such as those undertaken in the state of Amapá (see Adaime et al. 2023) or those resulting in the publications by Norrbom et al. (2015, 2021), using traps and fruit sampling, taking into consideration the seasonal occurrence of rainforest fruits. Nevertheless, sporadic collections have revealed the considerable diversity of *Anastrepha* species in Amazonas, which has the largest number of known species. Thus, it is not always necessary to venture deeply into the rainforest to ascertain the diversity of fruit flies. It is crucial that collections be methodical and continuous, as evidenced by the diversity of species, including two new to science, collected on the campus of the Federal University of Amazonas (UFAM) (Costa-Silva 2012), situated approximately 10 km from the Manaus city center.

In the present contribution, we describe two new species, review published distribution records, and provide a pictorial key to the 47 species known to occur in the state of Amazonas, Brazil.

## ﻿Materials and methods

### ﻿Identification

Females were identified with reference to the taxonomic keys provided by Zucchi (2000), Zucchi et al. (2011) and Norrbom et al. (2012), complemented by taxonomic works published after 2012 (Norrbom et al. 2015, 2021; Troya et al. 2020; Rodriguez and Norrbom 2021).

### ﻿Morphological study

Morphological terminology is based on White et al. (1999), wing venation on Cumming and Wood (2017), and wing bands on Stone (1942). Wing length was measured from the base of the costa to the wing apex in cell r_4+5_; wing width was measured from the apex of vein R_1_ to the posterior margin of cell m_4_. The width of cell r_4+5_ at the level of dm-m was measured on a line from the junction of dm-m and M_1_. The maximum width of cell r_4+5_ was measured perpendicular to vein M_1_ at the widest subapical part. The apical width of cell r_4+5_ was measured from the apex of vein R_4+5_ to the junction of M_1_ and the costa. The width of the distal part of the S-band was measured from the outer (anteroapical) margin of the costa to the inner (posterobasal) margin of the band perpendicular to the band at the apex of vein R_2+3_. The width of cell r_2+3_ was measured on the same straight perpendicular line from the apex of vein R_2+3_ to R_4+5_. Oviscape length was measured medially on the ventral side, from the ventromedial indentation to the apex. The aculeus tip length was measured ventrally from the sclerotized margin distal to the cloacal opening to the extreme apex. Illustrations of these measurements and the morphological characters described here can be seen in Norrbom et al. (2012). Type material is deposited at Museum of Entomology “Luiz de Queiroz” (MELQ), Department of Entomology and Acarology, Luiz de Queiroz College of Agriculture (ESALQ/USP).

### ﻿Images and measurements

The wings and aculei were photographed using a Leica DFC 450 camera coupled to an M205C stereomicroscope. The aculeus tip was photographed using a Zeiss Axio Imager 2 microscope. Measurements were taken using a Leica Wild M10 stereomicroscope. The images were enhanced using Corel Draw 2020 to adjust the color and implement minor corrections.

### ﻿Study site

The survey was conducted in an urban fragment of the Amazon Forest (3°05'87"S, 59°58'82"W), situated on the campus of the Federal University of Amazonas (UFAM) in Manaus, Amazonas, Brazil. This area encompasses 700 ha, with a perimeter of 12 km, elevation 46 m, and a predominant vegetation cover of dense ombrophilous forest. The campus is surrounded by urban infrastructure with dense human occupation and situated approximately 10 km from the city center of Manaus. The specimens were captured using McPhail traps baited with a 10% sugar cane solution and borax, hanged from trees at a height of 1.80 m above ground, from September 2010 to September 2011 (Costa-Silva 2012).

### ﻿License

The study was conducted with the approval of IBAMA (permit number 44295131) and focused on the identification of fruit flies captured in the Brazilian Amazon.

## ﻿Results

The description of the two new species brings the total number of *Anastrepha* species known in the state of Amazonas to 47, and in Brazil to 131 species. The *Anastrepha* species key for the state of Amazonas (Silva et al. 2023) has been amended and updated.

**Order Diptera Linnaeus, 1758**

**Family Tephritidae Newman, 1834**

### ﻿ Anastrepha dorsidentata

Taxon classificationAnimaliaDipteraTephritidae

Uramoto, Zucchi, Araújo & Savaris sp. nov.

DFA02C6E-91E1-5374-9D45-CCF95FB46951

https://zoobank.org/4867598B-F136-4162-82B7-713C016942AA

Figs 1–4

#### Type material.

***Holotype*** Brazil • 1 ♀; Amazonas, Manaus, campus of Federal University of Amazonas; 03°06'08.3"S, 59°58'31.6"W, 92 m elev.; collected on 15 July 2011, McPhail-type trap, food attractant, F.C. Costa-Silva leg. (MELQ ESALQENT001840).

#### Diagnosis.

*Anastrephadorsidentata* sp. nov. can be distinguished from other species of *Anastrepha* by the following combination of characters: scutum posteriorly without brown markings, subscutellum and mediotergite yellow to orange medially, dark brown laterally; wing pattern with C, S and V-bands complete; S-band middle section predominantly or entirely orange, with brown margins, V-band distal arm not connected to proximal arm of V-band along vein R_4+5_ and to S-band along vein R_2+3_; oviscape 1.63 mm long, aculeus 1.43 mm long, tip 0.18 mm long, gradually tapered with medial constriction, serrated part 0.84 length of tip, with serrations extending onto dorsal side basally. In the key of Zucchi (2000), *A.dorsidentata* sp. nov. runs to *A.sororcula* Zucchi, from which it differs in having the serrated part 0.84 times length of tip (0.45–0.70 in *A.sororcula*) and serrations extending onto the dorsal side basally. In the interactive key of Norrbom et al. (2012), *A.dorsidentata* sp. nov. runs to *A.canalis* Stone, from which it differs in having serrations displaced dorsally, forming ridge separate from lateral margins. *Anastrephadorsidentata* sp. nov. differs from *A.compressa* Stone, which also has the aculeus tip with dorsal ridge, by having lateral margins of aculeus tip near straight (slightly convex in *A.compressa*), triangular and distal arm of V-band not connected to proximal arm. *Anastrephareichardti* Zucchi also has an aculeus tip similar to that of *A.dorsidentata* sp. nov., but without a dorsal ridge.

#### Description.

Mostly orange. Setae dark red-brown.

***Head*.** Yellow to orange except brown ocellar tubercle. 3 frontal setae; 2 orbital setae. Ocellar seta weak, at most 1.5 times as long as ocellar tubercle. Facial carina, in profile, straight on dorsal two-thirds. Antenna not extended to ventral facial margin. Palpus in lateral view dorsally curved, evenly setulose. Face with ventral part gradually tapering laterally.

***Thorax*.** Mostly orange; without brown markings, scuto–scutellar suture without brown spot; with following areas white to pale yellow: postpronotal lobe and lateral margin of scutum bordering it; sublateral scutal vitta from transverse suture to posterior margin, including base of intra-alar seta; medial scutal vitta present with posterior end ovoid; scutellum, dorsal margins of anepisternum and katepisternum; katepimeron, most of anatergite and katatergite. Subscutellum and mediotergite dark brown laterally, yellow to orange medially. Mesonotum 2.38 mm long. Postpronotal lobe, notopleuron, scutum, and scutellum entirely microtrichose; scutal setulae golden yellow to orange. Chaetotaxy typical for genus. Katepisternal seta orange-brown, much weaker than and less than half as long as anepisternal seta.

***Legs*.** Entirely yellow to orange.

**Figures 1–4.**
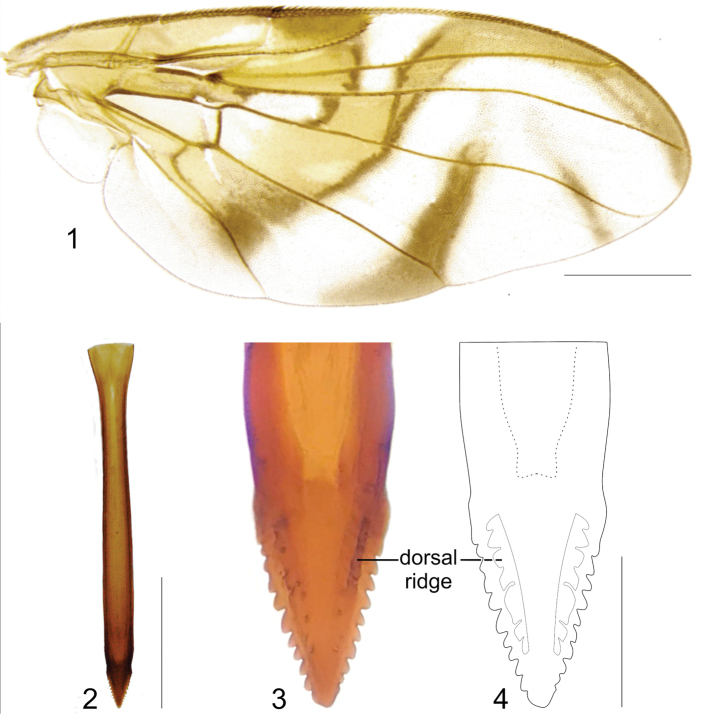
*Anastrephadorsidentata* sp. nov. **1.** Wing; **2.** Aculeus (ventral view); **3.** Aculeus tip (dorsal view); **4.** Aculeus tip showing dorsal ridge (dorsal view). Photographs by Alexandre S. (wing), Keiko Uramoto (aculeus and aculeus tip); illustration by M. Savaris. Scale bars: 1.0 mm (wing and aculeus); 0.1 mm (aculeus tip).

***Wing*.** Length 6.45 mm, width 2.61 mm, ratio 2.47. Apex of vein R_1_ at 3.27 wing length, proximal to level of anterior end of crossvein r-m. Cell c 1.18 times as long as pterostigma; pterostigma 1.45 times as long as wide. Vein R_2+3_ not sinuous. Crossvein r-m at 0.65 distance from bm-m to dm-m on vein M_1_. Vein M_1_ moderately curved apically; cell r_4+5_ at apex 1.05 times as wide as at level of dm-m, 0.81 times as wide as maximum subapical width. Cell cua with distal lobe relatively short, cua 1.5 times as long as anterior margin, lobe 0.58 times as long as vein CuA+CuP. Wing pattern (Fig. 1) mostly orange and moderate brown. C-band mostly orange, most of cell c sometimes paler but without subapical hyaline area, most of pterostigma orange-brown, distal margin in cells r_1_ and r_2+3_ narrowly brown, fork of vein Rs with ovoid brown spot, junction of costa and crossvein h without brown spot, and cell br with brown mark anterior to proximal end of cell bm and small brown mark on apical margin of band bordering vein R_4+5_. C-band and S-band connected along vein R_4+5_. Basal hyaline area in cell dm relatively small, occupying less than one-third of cell. Cell bm hyaline, microtrichose only on subapical fold. Basal half of S-band mostly orange, anterobasal margin narrowly brown except in cell dm, posterodistal margin narrowly brown, more broadly in cell m_4_, but at most extending to apex of lobe of cell cua, margin with a small incision in cell m_4_; distal section narrowly brown on most of posterior margin and in cell r_4+5_; moderately broad, at apex of vein R_2+3_, 0.53 times width of cell r_2+3_, without marginal hyaline areas; hyaline area proximal to apex of band extending nearly to vein R_2+3_. V-band with proximal arm brown in cell m_4_ and on most proximal and distal margins; separated from S-band along vein R_4+5_; on posterior margin extending two-thirds of distance to vein CuA+CuP; distal arm mostly brown, not connected to proximal arm.

***Abdomen*.** Mostly orange, without brown markings.

***Female terminalia*.** Oviscape 1.63 mm long, 0.68 times as long as mesonotum, straight in lateral view; entirely orange to pale brown; spiracle at basal 0.34. Eversible membrane not dissected, pattern of dorsobasal denticles not visible. Aculeus slightly ventrally curved in lateral view, 1.43 mm long, 0.87 times oviscape length; in ventral view base expanded, triangular, 0.22 mm wide, shaft 0.12 mm wide at midlength (Fig. 2); tip 0.18 mm long, 0.12 times aculeus length, 0.09 mm wide at base, 0.11 mm wide preapically, 1.62 times as long as preapical width; in ventral view (Figs 3, 4) gradually tapered, moderately constricted before serrated part, serrate part triangular, 0.84 times tip length, with serrations extending to dorsal side basally. Spermathecae not dissected.

**Male.** Unknown.

#### Distribution.

*Anastrephadorsidentata* sp. nov. is known only from Manaus, Amazonas, Brazil.

#### Biology.

The host plants and other aspects of the biology of this species are unknown.

#### Etymology.

The species epithet is a Latin adjective formed by *dorsum* (dorsal) and *dentatus* (toothed), with reference to the dorsal position of some teeth at the aculeus tip.

#### Comments.

*Anastrephadorsidentata* sp. nov. belongs to the *fraterculus* group, on the basis of its aculeus tip and the brown lateral markings on the subscutellum and mediotergite.

### ﻿ Anastrepha norrbomi

Taxon classificationAnimaliaDipteraTephritidae

Uramoto, Zucchi, Araújo & Savaris sp. nov.

E5762E1D-68CA-51DA-908C-2FD56BEA3AD3

https://zoobank.org/B8602B9B-207F-468D-B039-EFFA19109C1E

Figs 5–7

#### Type material.

***Holotype*** Brazil • 1 ♀; Amazonas, Manaus, campus of Federal University of Amazonas; 03°05'51.1"S, 59°58'23.8"W; 92 m elev.; collected on 15 July 2011, McPhail-type trap, food attractant, F.C. Costa-Silva leg. (MELQ ESALQENT001841).

#### Diagnosis.

*Anastrephanorrbomi* sp. nov. can be recognized by the following combination of characters: face with ventral part gradually tapered laterally; scutal and scutellar setae well developed, dark brown to black, mediotergite and subscutellum entirely yellow, without brown marks; wing pattern with C- and S-bands complete, distal arm of V-band absent; oviscape 4.72 mm long, 1.18 as long as mesonotum; aculeus tip short (0.07 mm), 0.01 times aculeus length, subquadrate (0.83 times as long as preapical width), nonserrate and blunt. In the key of Zucchi (2000), *A.norrbomi* sp. nov. runs to *A.quararibae* Lima, from which it differs in having the aculeus tip much shorter (0.25–0.33 mm in *A.quararibae*) and the distal arm of the V-band absent. In the key of Steyskal (1977), it runs to *A.panamensis* Greene, but that species has the distal arm of the V-band connected to the proximal arm and a shorter aculeus (2.65–3.35 mm) with a tapered, triangular tip.

#### Description.

Mostly orange. Setae dark brown to black.

***Head*.** Yellow to orange except brown ocellar tubercle. 4 frontal setae; 2 orbital setae. Ocellar seta weak, at most 1.5 times as long as ocellar tubercle. Facial carina, in profile, straight on dorsal two-thirds. Antenna not extending to ventral facial margin. Palpus in lateral view dorsally curved, evenly setulose. Face with ventral part gradually tapering laterally.

***Thorax*.** Mostly orange; without brown marking, scuto–scutellar suture without brown spot; with following areas white to pale yellow: postpronotal lobe and lateral margin of scutum bordering it; medial scutal vitta present with posterior end ovoid; sublateral scutal vitta from transverse suture to posterior margin, including base of intra-alar seta; scutellum, dorsal margins of anepisternum and katepisternum; katepimeron, most of anatergite and katatergite. Subscutellum and mediotergite entirely orange. Mesonotum 3.98 mm long. Postpronotal lobe, notopleuron, scutum, and scutellum entirely microtrichose; scutal setulae yellow to orange. Chaetotaxy typical for genus. Katepisternal seta orange, much weaker than and less than half as long as anepisternal seta.

***Legs*.** Entirely yellow to orange.

**Figures 5–7.**
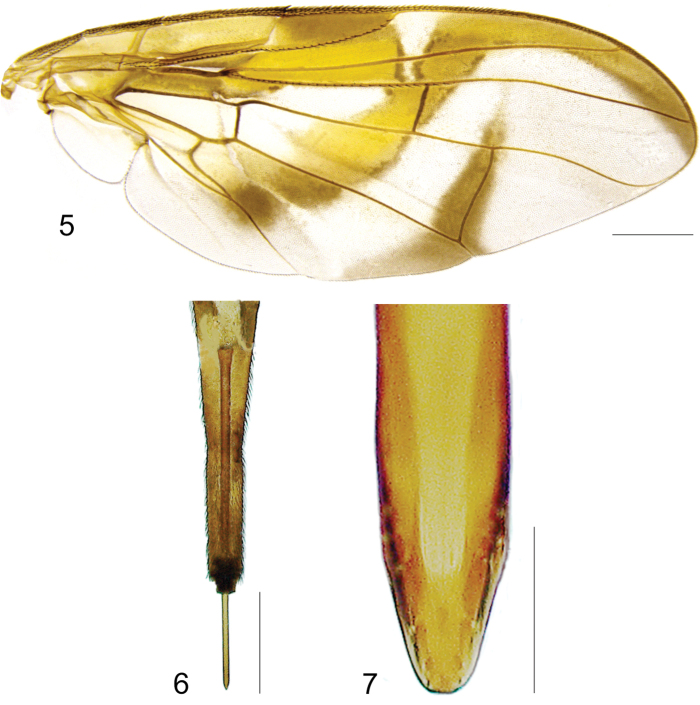
*Anastrephanorrbomi* sp. nov. **5.** Wing; **6.** Oviscape and aculeus (ventral view); **7.** Aculeus tip (ventral view). Photographs by Alexandre S. Araújo (Wing), Keiko Uramoto (Aculeus and aculeus tip). Scale bars: 1.0 mm (wing and aculeus); 0.1 mm (aculeus tip).

***Wing*.** Length 8.45 mm, width 3.36 mm, ratio 2.51. Apex of vein R_1_ at 0.55 wing length, proximal to level of anterior end of crossvein r-m. Cell c 1.27 times as long as pterostigma; pterostigma 3.60 times as long as wide. Vein R_2+3_ not sinuous. Crossvein r-m at 0.69 distance from bm-m to dm-m on vein M_1_. Vein M_1_ moderately curved apically; cell r_4+5_ at apex 0.82 times as wide as at level of dm-m, 0.73 times as wide as maximum subapical width. Cell cua with distal lobe relatively short, length of cua 1.50 times as long as anterior margin, lobe 0.52 times as long as vein CuA+CuP. Wing pattern (Fig. 5) mostly orange and moderate brown. C-band mostly orange, most of cell c sometimes paler but without subapical hyaline area, most of pterostigma orange-brown, distal margin in cells r_1_ and r_2+3_ narrowly brown, fork of vein Rs with ovoid brown spot, junction of costa and crossvein h with brown spot, and cell br with small ovoid brown mark on apical margin of band bordering vein R_4+5_. C-band and S-band connected along vein R_4+5_. Basal hyaline area in cell dm relatively small, occupying less than one-third of the cell. Cell bm hyaline, microtrichose only on subapical fold. Basal half of S-band mostly orange, anterobasal margin narrowly brown except in cell dm, posterodistal margin narrowly brown, more broadly in cell m_4_, but at most extending to apex of lobe of cell cua, margin with incision in cell m_4_; distal section narrowly brown on most of posterior margin and in cell r_4+5_; moderately broad, at apex of vein R_2+3_, 0.57 times width of cell r_2+3_, without marginal hyaline areas; hyaline area proximal to apex of band extending nearly to vein R_2+3_. V-band with proximal arm brown in cell m_4_ and on most of proximal and distal margins; separated from S-band along vein R_4+5_; on posterior margin extending two-thirds of distance to vein CuA+CuP; distal arm absent.

***Abdomen*.** Mostly orange, without brown markings.

***Female terminalia*.** Oviscape 4.72 mm long, 1.18 times as long as mesonotum, straight in lateral view; entirely orange to pale brown; spiracle at basal 0.26. Eversible membrane not dissected, pattern of dorsobasal denticles not visible. Aculeus slightly ventrally curved in lateral view, 4.27 mm long, 0.90 times oviscape length; in ventral view base expanded, triangular, 0.21 mm wide, shaft 0.08 mm wide at midlength (Fig. 6); tip 0.07 mm long, 0.01 times aculeus length, 0.07 mm wide at base, 0.08 mm wide preapically, 0.83 times as long as preapical width; in ventral view slightly tapered, non-serrate, lateral margin straight, tip truncated (Fig. 7). Spermathecae not dissected.

***Male terminalia*.** Unknown.

#### Distribution.

*Anastrephanorrbomi* sp. nov. is known only from Manaus, Amazonas, Brazil.

#### Biology.

The host plants and other aspects of the biology of this species are unknown.

#### Etymology.

The species epithet pays tribute to Allen L. Norrbom, for his notable contributions to knowledge of the diversity of the genus *Anastrepha*, describing numerous species, and for revealing several morphological characters that allowed the standardization of species descriptions, leading to the development of an interactive illustrated key for hundreds of *Anastrepha* species.

#### Comments.

This species has not been placed in a species group.

## ﻿Discussion

The records of *Anastrepha* species in the state of Amazonas were obtained by multiple researchers over approximately 90 years (see Zucchi and Moraes 2025). The first record of the genus in the state (*A.fenestrata* Lutz & Lima) was published in the 1910s (Lutz and Lima 1918), while the most recent record (*A.caballeroi* Norrbom) was reported by Uramoto et al. (2024). Descriptions of new species from the state of Amazonas have been published intermittently (Lima 1934; Stone 1942; Norrbom 1991; Norrbom and Korytkowski 2009, 2011, 2012; Norrbom et al. 2012, 2015, 2021).

The first key for identifying *Anastrepha* species from the Brazilian Amazon included 32 species and data from five states of the North region: Amapá (3 species), Amazonas (25), Pará (15), Rondônia (5), and Roraima (8) (Silva and Ronchi-Teles 2000). At that time, no records were available for the northern states of Acre and Tocantins. Approximately a decade later, an illustrated key was produced for 54 species of *Anastrepha* from the Amazon region, including records from all nine states of the region (Zucchi et al. 2011). A key was recently published exclusively for species native to the state of Amazonas (Silva et al. 2023). This key is currently being amended and updated, with the inclusion of *A.caballeroi* Norrbom, *A.cruzi* Lima, *A.dorsidentata* sp. nov., and *A.norrbomi* sp. nov.

**Figure 8.**
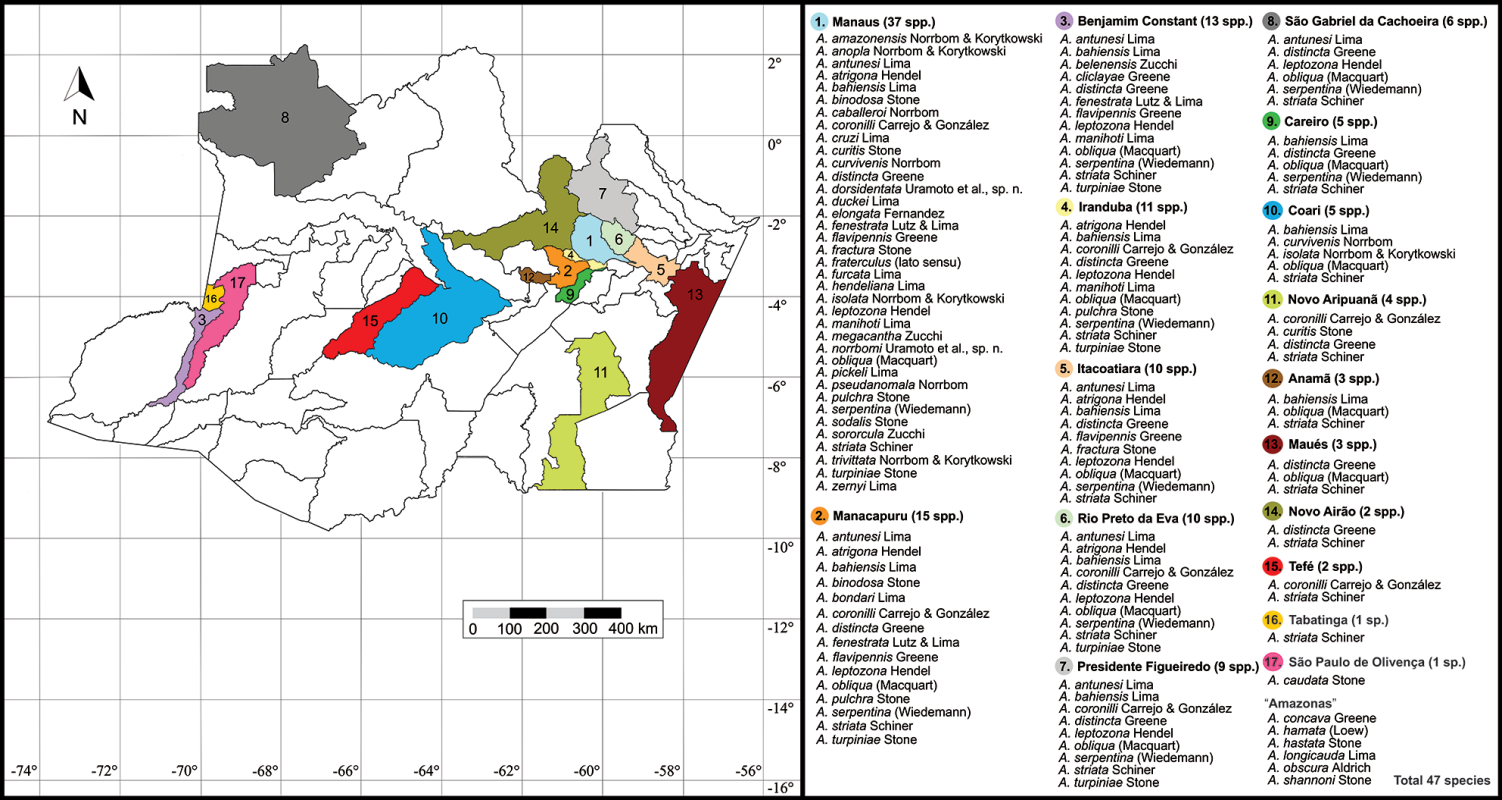
Distribution of *Anastrepha* species in the municipalities of the state of Amazonas, Brazil. Illustration by M. Savaris.

The state of Amazonas comprises 62 municipalities, yet only 17 of these have documented records of *Anastrepha* species. About 80% (37) of the species were recorded in Manaus. In contrast, records of *Anastrepha* species in municipalities in the southern part of the state, in the region of the deforestation arc, are scarce (Silva et al. 2023) (Fig. 8). *Anastrephastriata* Schiner appears to be the most widely distributed species in the state, having been recorded in 16 municipalities, followed by *A.distincta* Greene and *A.obliqua* (Macquart) (both in 12). Nevertheless, 22 species were documented in a single municipality (Table 1). The considerable number of municipalities with only a single recorded species suggests that collections in the state have been sporadic or occasional. Four species (*A.amazonensis* Norrbom, *A.cruzi* Lima, *A.megacantha* Zucchi, and *A.trivittata* Norrbom) are known only from the state of Amazonas. *Anastrephacaudata* Stone, originally described from the Amazon, is likely to occur in Colombia as well (Norrbom 2025). For four species (*Anastrephahamata* (Loew), *A.hastata* Stone, *A.obscura* Aldrich, and *A.shannoni* Stone) recorded from Amazonas the municipality is unknown, the sites of occurrence were given only as Amazonas. Conversely, *A.grandicula* Norrbom and *A.macracantha* Norrbom & Korytkowski were erroneously recorded in the state of Amazonas (Zucchi 2007; Silva et al. 2023, respectively). *Anastrephagrandicula* has been recorded only from Colombia (Amazon Basin, Rio Putumayo, Puerto Arica) and Peru (Junín) (Norrbom 1991; Mengual et al. 2017), and *A.macracantha* occurs in eastern Ecuador (Orellana, Sucumbíos) (Norrbom and Korytkowski 2012). On the other hand, *A.concava* Greene was collected in the city of São Paulo de Olivença, state of Amazonas (Norrbom and Caraballo 2003), but was incorrectly recorded for the state of São Paulo (Stone 1942). In addition to Brazil (Amazonas and Pará), *A.concava* also occurs in Bolivia, Costa Rica, Ecuador, Panama, and Peru (Norrbom 2025). Host plants are unknown for about 80% of the *Anastrepha* species from Amazonas (Table 1) (see Zucchi and Moraes 2025).

**Table 1.** Distribution of *Anastrepha* species in the state of Amazonas, Brazil.

Species of *Anastrepha*	Municipalities (for numbers see Fig. 8)																
	1	2	3	4	5	6	7	8	9	10	11	12	13	14	15	16	17
*А.amazonensis* ^1^	X																
*А.anopla*	X																
*А.antunesi* ^2^	X	X	X		X	X	X	X									
*А.atrigona* ^2^	X	X		X	X	X											
*А.bahiensis* ^2^	X	X	X	X	X	X	X		X	X		X					
*А.belenensis*			X														
*А.binodosa*	X	X															
*А.bondari* ^2^		X															
*А.caballeroi*	X																
*А.caudata*																	X
*А.chiclayae*			X														
*А.concava* ^3^	–	–	–	–	–	–	–	–	–	–	–	–	–	–	–	–	–
*А.coronilli* ^2^	X	X		X		X	X				X				X		
*А.cruzi* ^1^	X																
*А.curitis* ^2^	X										X						
*А.curvivenis* ^1^	X									X							
*А.distincta* ^2^	X	X	X	X	X	X	X	X	X		X		X	X			
*А.dorsidentata* ^1^	X																
*А.duckei* ^2^	X																
*А.elongata*	X																
*А.fenestrata*	X	X	X														
*А.flavipennis* ^2^	X	X	X		X												
*А.fractura* ^2^	X				X												
*А.fraterculus*	X																
*А.furcata*	X																
*А.hamata* ^3^	–	–	–	–	–	–	–	–	–	–	–	–	–	–	–	–	–
*А.hastata* ^3^	–	–	–	–	–	–	–	–	–	–	–	–	–	–	–	–	–
*А.hendeliana*	X																
*А.isolata*	X									X							
*А.leptozona* ^2^	X	X	X	X	X	X	X	X									
*А.longicauda* ^3^	–	–	–	–	–	–	–	–	–	–	–	–	–	–	–	–	–
*А.manihoti* ^2^	X		X	X													
*А.megacantha* ^1^	X																
*А.norrbomi* ^1^	X																
*А.obliqua* ^2^	X	X	X	X	X	X	X	X	X	X		X	X				
*А.obscura* ^1, 3^	–	–	–	–	–	–	–	–	–	–	–	–	–	–	–	–	–
*А.pickeli* ^2^	X																
*А.pseudanomala*	X																
*А.pulchra* ^2^	X	X		X													
*А.serpentina* ^2^	X	X	X	X	X	X	X	X	X								
*А.shannoni* ^3^	–	–	–	–	–	–	–	–	–	–	–	–	–	–	–	–	–
*А.sodalis*	X																
*А.sororcula*	X																
*А.striata* ^2^	X	X	X	X	X	X	X	X	X	X	X	X	X	X	X	X	
*А.trivittata* ^1^	X																
*А.turpiniae* ^2^	X	X	X	X		X	X										
*А.zernyi*	X																

**^1^**Recorded only from Amazonas state; **^2^**Known hosts in Amazonas state; **^3^**Unknown locality.

A compilation of the records, which encompass a wide temporal and geographic scope, indicates that collections of *Anastrepha* species in the state of Amazonas have been sporadic and concentrated mostly in the vicinity of urban centers. The factors that may explain this limited collection effort are the same as for the entire Brazilian Amazon, namely the vast territorial extent, inaccessible locations, and a scarcity of human resources for conducting entomological studies (Sousa et al. 2021).

Notwithstanding these constraints, Amazonas is the Brazilian state with the most records of *Anastrepha* species. However, knowledge of the diversity of fruit flies in Amazonas remains at a very incipient level. This reasoning is supported by data collected on the UFAM campus (fragment of urban rainforest), where 18 species of *Anastrepha* were collected in McPhail traps over a 13-month period, in addition to other unidentified species (Costa-Silva 2012). These species were subsequently identified, resulting in new records for the state of Amazonas (Costa-Silva et al. 2020), including the rediscovery of *A.cruzi* Lima, 80 years after its original description (Uramoto et al. 2024), and the discovery of two new species (*A.dorsidentata* sp. nov. and *A.norrbomi* sp. nov.) described here. In this rainforest fragment, where numerous fruit species typical of the Amazon region are present, nearly half of the species so far recorded in the state of Amazonas were collected. This finding underscores the importance and necessity of continuous and frequent surveys to assess the diversity of fruit flies in the Amazon. Such surveys would increase the probability of collecting more species of fruit flies, given the seasonality of fruits in the Amazon region, which is home to a vast array of native fruit species (Cavalcante 2010). Consequently, in view of the logistical challenges associated with collecting in remote locations, continuous sampling (utilizing traps and/or collecting fruits), even in forest fragments situated near urban centers, can be of great value in understanding the diversity of *Anastrepha* fruit flies in the Amazon biome. On the other hand, the enormous biodiversity of the rainforest reminds us of the words of Isaac Newton, broadly paraphrased: “…we collect one species or another, while the enormous diversity of species in the Amazon Rainforest remains unknown to us”.

### ﻿Illustrated key to the species of *Anastrepha* (female) recorded in the state of Amazonas, Brazil

**Table:** 

1	C-band and S-band fused, covering anterior margin of wing	**2**
1′	C-band and S-band at least partially separated by hyaline area distal to apex of vein R_1_	**4**
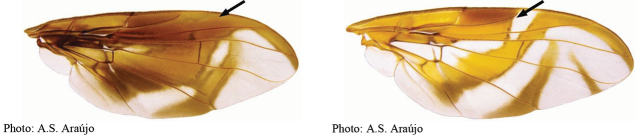		
2(1)	Cell br with hyaline area extending at least one third of cell length	***A.trivittata* Norrbom & Korytkowski**
2′	Cell br without hyaline area	**3**
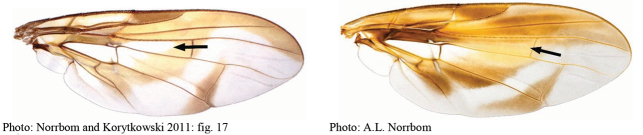		
3(2′)	Cell r_2+3_ with subapical hyaline area extending to vein R_2+3_; V-band with proximal arm often partially joined to S-band in cell dm; aculeus tip not serrated	***A.atrigona* Hendel**
3′	Cell r_2+3_ entirely infuscated; V-band with proximal arm separated from S-band in cell dm; aculeus tip serrated	***A.shannoni* Stone**
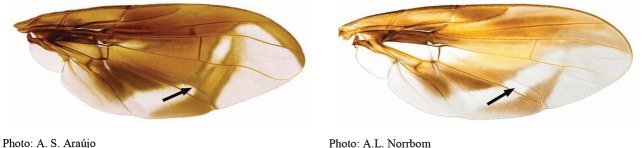		
4(1′)	Eversible membrane with dorsobasal denticles all small and weakly developed; aculeus width less than 0.05 mm	**5**
4′	Eversible membrane with dorsobasal denticles all sclerotized; aculeus width more than 0.05 mm	**8**
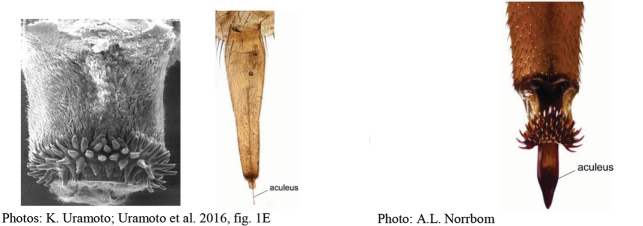		
5(4)	C, S and V-bands broadly fused	***A.obscura* Aldrich**
5′	C-, S- and V-bands at least partially separated	**6**
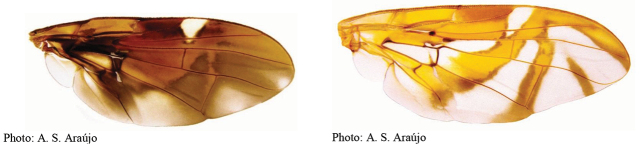		
6(5′)	Aculeus 6–8 mm long; tip nonserrate	***A.longicauda* Lima**
6′	Aculeus less than 5 mm long; tip serrate	**7**
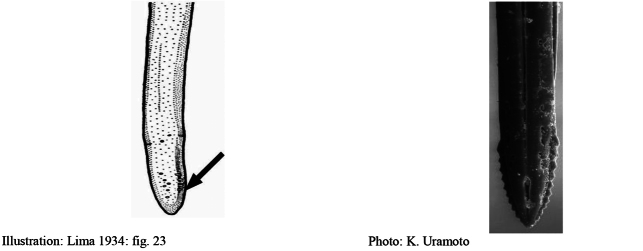		
7(6′)	Aculeus tip with triangular, acute apex	***A.hamata* Loew**
7′	Aculeus tip with blunt apex	***A.zernyi* Lima**
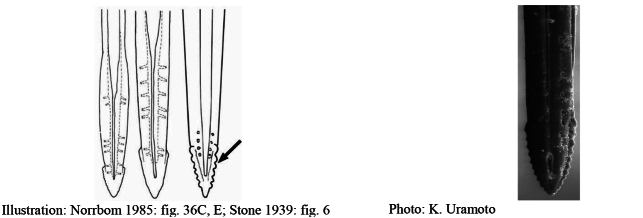		
8(4′)	Eversible membrane with one very large slender medial hook-like denticle much larger than others; C-, S- and V-bands separated; oviscape 10 mm long	***A.megacantha* Zucchi**
8′	Eversible membrane with multiple large equal to subequal hook-like denticles	**9**
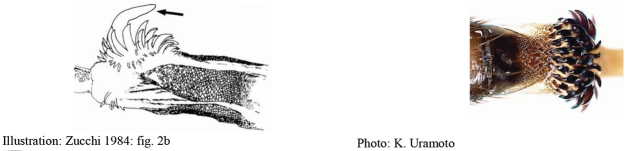		
9(8′)	Mesonotum, excluding white to yellow vittae, mostly dark brown	**10**
9′	Mesonotum, excluding vittae, mostly yellowish to orange	**12**
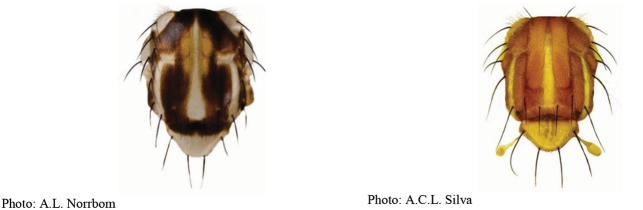		
10(9)	Basal hyaline area between C-band and S-band absent	***A.pulchra* Stone**
10′	Basal hyaline area between C-band and S-band extending into cell br but not touching vein R_4+5_	**11**
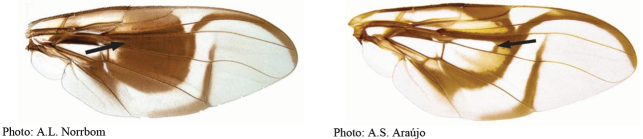		
11(10′)	Abdominal tergites brown with T-shaped yellow or white mark; aculeus length 2.55–3.85 mm	***A.serpentina* (Wiedemann)**
11′	Abdominal tergites with brown vittae; aculeus length 3.95–4.35 mm	***A.pseudanomala* Norrbom**
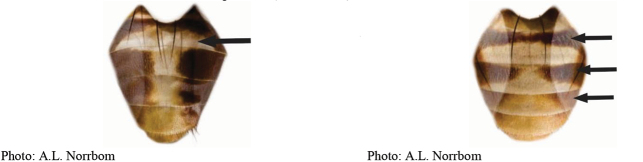		
12(9′)	Oviscape spiracle near base (at basal 0.06–0.15 of length); aculeus tip with constriction, more than apical half serrate	***A.curitis* Stone**
12′	Oviscape spiracle far from base (distal to basal 0.15)	**13**
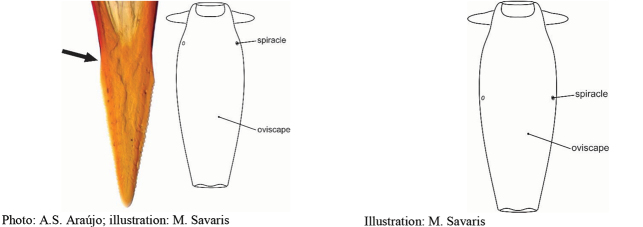		
13(12′)	Scutum with U-shaped dark brown mark interrupted at transverse suture; aculeus length 1.95–2.3 mm	***A.striata* Schiner**
13′	Scutum without longitudinal dark vittae	**14**
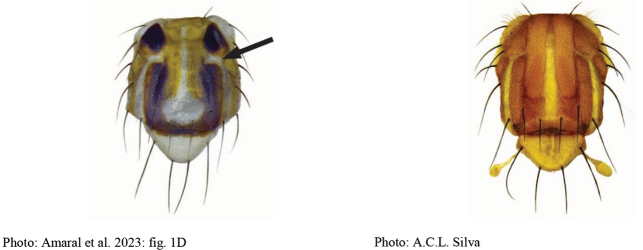		
14(13′)	Wing bands mostly dark brown	**15**
14′	Wing bands predominantly light brown and/or orange	**21**
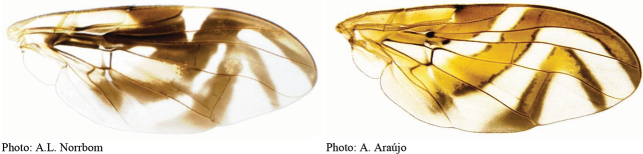		
15(14)	Cell br entirely infuscate, without hyaline areas	***A.amazonensis* Norrbom & Korytkowski**
15′	Cell br not entirely infuscate, with subapical hyaline area	**16**
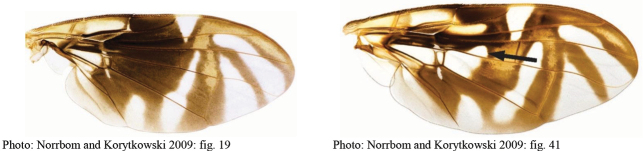		
16(15′)	S-band with width of basal portion less than half length of CuA+CuP	**17**
16′	S-band with width of basal portion more than half length of CuA+CuP	**18**
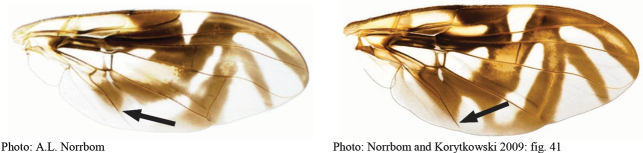		
17(16)	Aculeus length: 5–6 mm; aculeus tip 0.1–0.11 mm wide	***A.hendeliana* Lima**
17′	Aculeus length: 3.3–3.8 mm; aculeus tip 0.14–0.16 mm wide	***A.caudata* Stone**
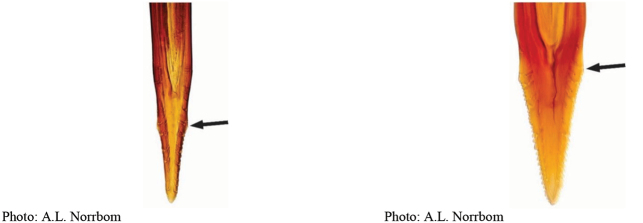		
18(16′)	V-band connected to S-band at two points	***A.isolata* Norrbom & Korytkowski**
18′	V-band connected to S-band at a single point	**19**
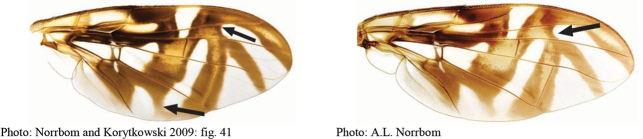		
19(18′)	Base of S-band with posterior extension to almost to wing margin in middle of cell m_4_; aculeus tip with medium-sized serrations	***A.fenestrata* Lutz & Lima**
19′	Base of S-band without posterior extension in middle of cell m_4_	**20**
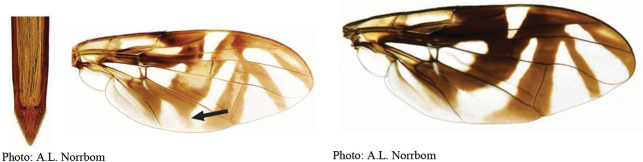		
20(19′)	Vein M_1_ very strongly curved apically; aculeus strongly curved in lateral view, 1.19–2.27 mm long	***A.furcata* Lima**
20′	Vein M_1_ slightly curved apically; aculeus straight in lateral view, 6.4–9.20 mm long	***A.concava* Greene**
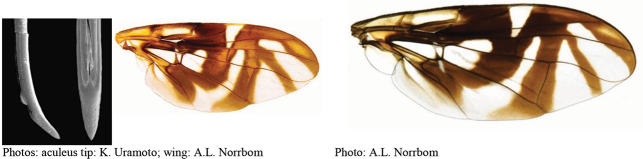		
21(14′)	Mediotergite not darkened laterally	**22**
21′	Mediotergite dark brown laterally	**40**
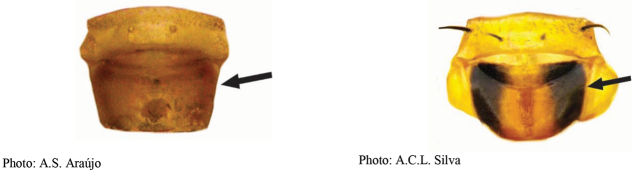		
22(21′)	Aculeus tip with preapical constriction; eversible membrane with very long pattern of small denticles, platelike, not hook-like	***A.anopla* Norrbom & Korytkowski**
22′	Aculeus tip without preapical constriction; eversible membrane with hook-like denticles	**23**
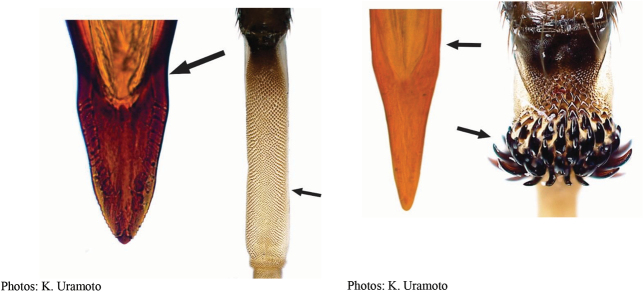		
23(22′)	Aculeus tip not serrate	**24**
23′	Aculeus tip serrate	**28**
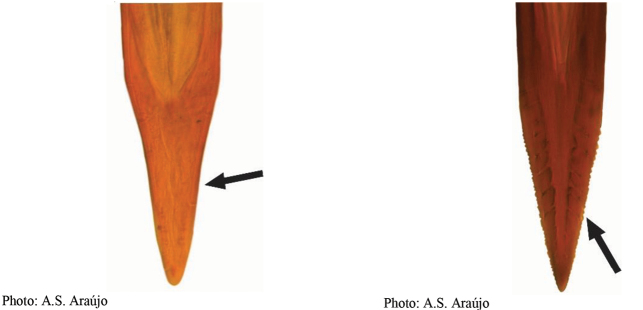		
24(23)	Aculeus tip with lateral projections on basal half	***A.hastata* Stone**
24′	Aculeus tip without lateral projection	**25**
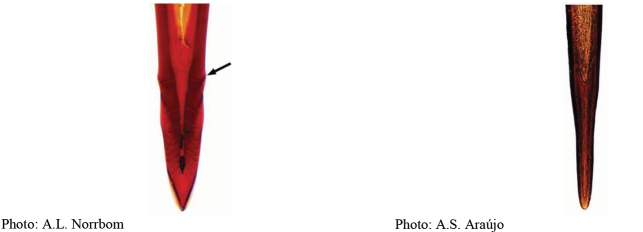		
25(24′)	Vein M_1_ strongly curved apically	***A.curvivenis* Norrbom**
25′	Vein M_1_ slightly curved apically	**26**
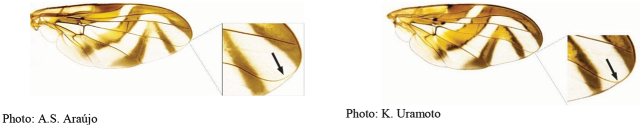		
26(25′)	Aculeus tip very short (0.07 mm long)	***A.norrbomi* Uramoto et al. sp. nov.**
26′	Aculeus tip long (0.20–0.37 mm)	**27**
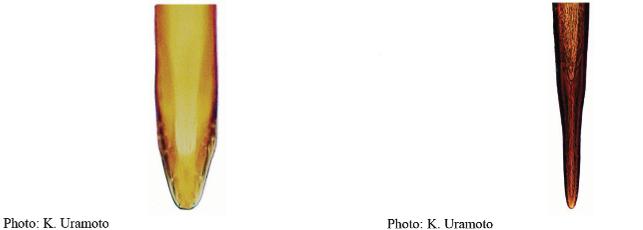		
27(26′)	C- and S-bands connected; aculeus 0.12–0.14 mm wide	***A.bondari* Lima**
27′	C- and S-bands disconnected; aculeus tip 0.05–0.09 mm wide	***A.caballeroi* Norrbom**
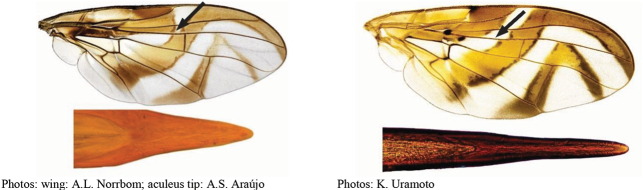		
28(23′)	Aculeus tip with a constriction basal to serrate part and with medium-sized widely spaced serrations	***A.cruzi* Lima**
28′	Aculeus tip without constriction basal to serrate part and with minute, closely spaced serrations	**29**
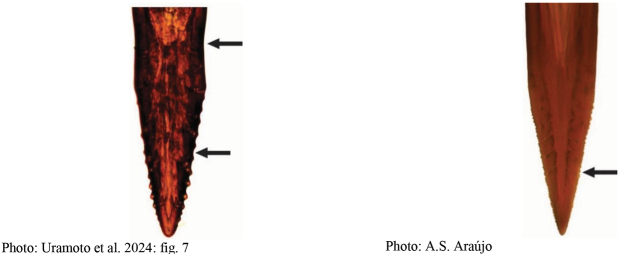		
29(28′)	Aculeus tip with serrations on less than apical half	**30**
29′	Aculeus tip with serrations on at least apical half	**31**
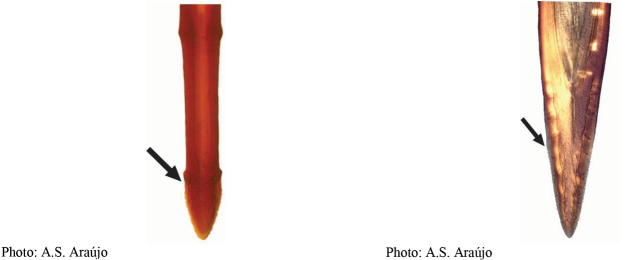		
30(29)	Aculeus 9.25–12.25 mm long; aculeus tip without lateral projections	***A.elongata* Fernandez**
30′	Aculeus 4.10–5.20 mm long; aculeus tip with lateral projections	***A.binodosa* Stone**
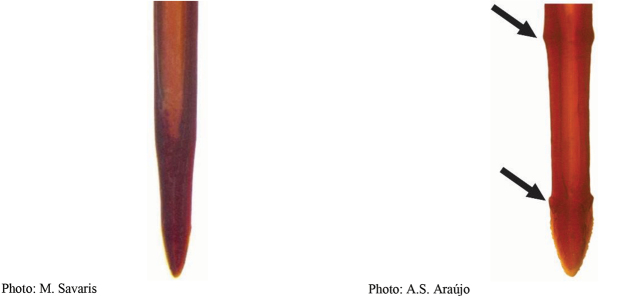		
31(29′)	C- and S-bands separate	**32**
31′	C- and S-bands connected	**33**
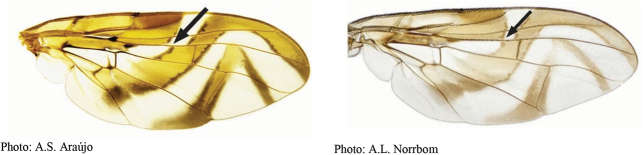		
32(31)	Vein M_1_ slightly curved apically	***A.chiclayae* Greene**
32′	Vein M_1_ strongly curved apically	***A.leptozona* Hendel**
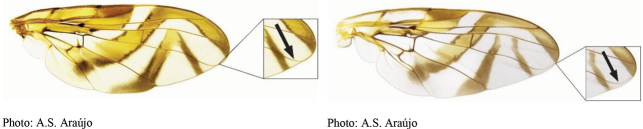		
33(31′)	Aculeus tip tapering abruptly	***A.fractura* Stone**
33′	Aculeus tip not tapering abruptly	**34**
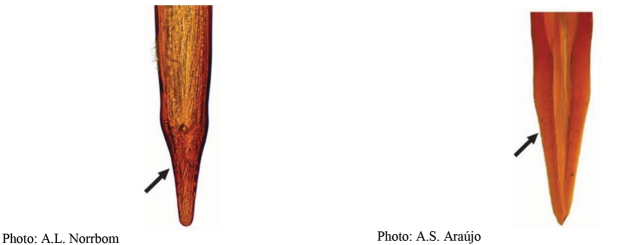		
34(33′)	Aculeus tip serrate	**35**
34′	Aculeus tip not serrate	**39**
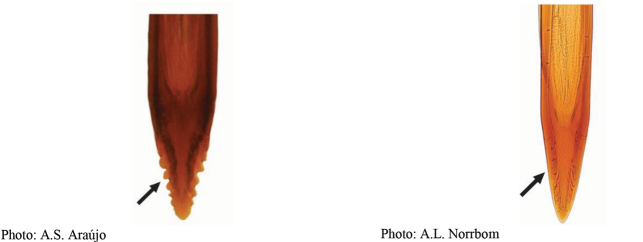		
35(34)	Aculeus tip with serrations beyond level of cloacal opening	**36**
35′	Aculeus tip with serrations not reaching cloacal opening	**37**
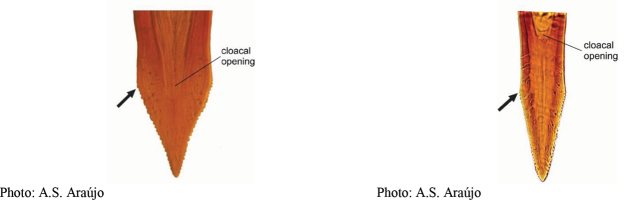		
36(35)	Aculeus tip with a slight constriction before serration	***A.manihoti* Lima**
36′	Aculeus tip with no constriction before serration	***A.pickeli* Lima**
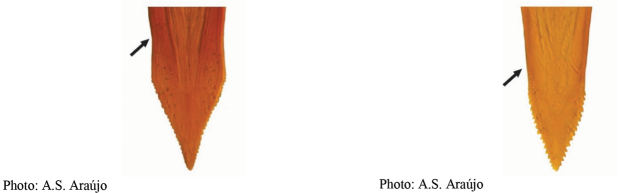		
37(35′)	Aculeus tip with few large serrations	***A.antunesi* Lima**
37′	Aculeus tip with many small to tiny serrations	**38**
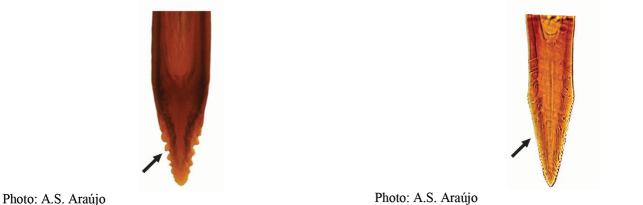		
38(37′)	S- and V-bands not connected; aculeus tip with distinct serrations	***A.duckei* Lima**
38′	S- and V-bands connected; aculeus tip with fine serrations	***A.sodalis* Stone**
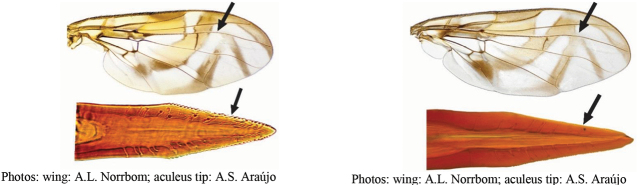		
39(34′)	Cell bm yellowish; aculeus tip without constriction	***A.flavipennis* Greene**
39′	Cell bm hyaline; aculeus tip with constriction	***A.belenensis* Zucchi**
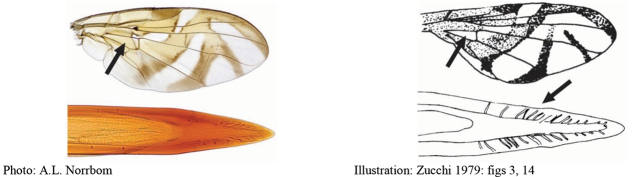		
40(21′)	Aculeus tip with serrations extending at least to half tip length	**41**
40′	Aculeus tip with serrations along less than half tip length	**45**
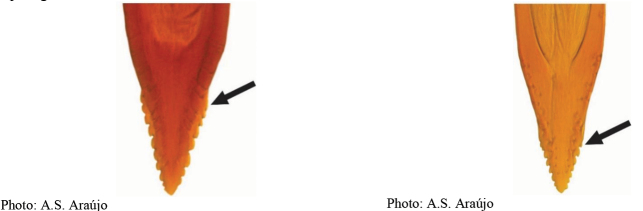		
41(40)	Aculeus tip with distinct constriction before serrations	**42**
41′	Aculeus tip with slight constriction or with no constriction before serrations	**44**
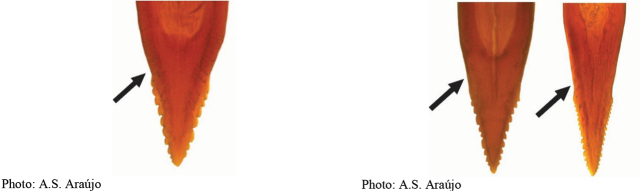		
42(41)	Aculeus tip with dorsal ridge	***A.dorsidentata* Uramoto et al. sp. nov.**
42′	Aculeus tip without dorsal serrations	**43**
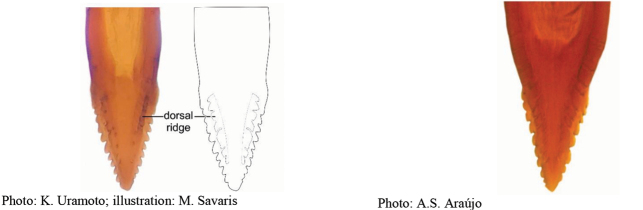		
43(42′)	Aculeus tip long (tip length/tip width at base about 1.9	***A.fraterculus* (Wiedemann)**
43′	Aculeus tip short (tip length/ tip width at base about 1.4	***A.sororcula* Zucchi**
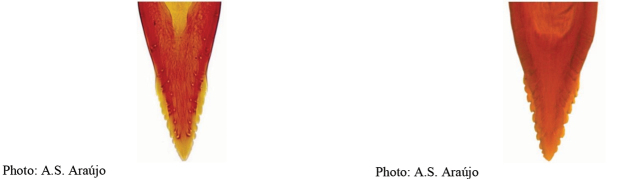		
44(41′)	Aculeus tip about 0.20 mm long; serrations acute	***A.obliqua* (Macquart)**
44′	Aculeus tip 0.27–0.30 mm long; serrations rounded	***A.turpinae* Stone**
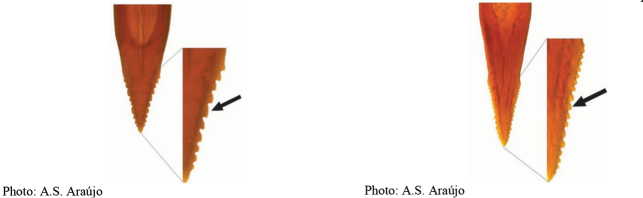		
45(40′)	Aculeus length less than 2.00 mm	***A.bahiensis* Lima**
45′	Aculeus length at least 2.00 mm	**46**
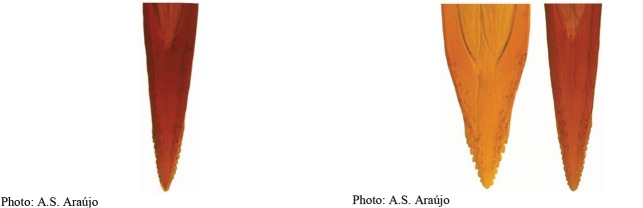		
46(45′)	Aculeus tip with prominent and acute serrations	***A.coronilli* Carrejo & González**
46′	Aculeus tip with slightly prominent and rounded serrations	***A.distincta* Greene**
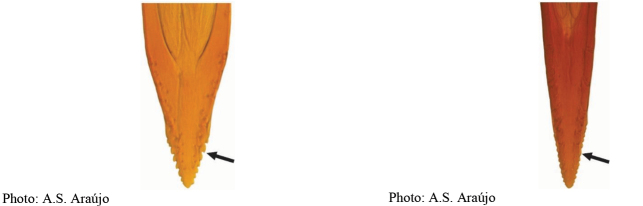		

## Supplementary Material

XML Treatment for Anastrepha dorsidentata

XML Treatment for Anastrepha norrbomi

## ﻿Additional information

### Use of AI

No use of AI was reported.

### Author ORCIDs

Keiko Uramoto https://orcid.org/0000-0003-2676-0035

Alexandre S. Araújo https://orcid.org/0000-0003-0245-763X

Francisco C. Costa-Silva https://orcid.org/0009-0006-4444-4684

Neliton M. Silva https://orcid.org/0000-0002-6812-729X

Marcoandre Savaris https://orcid.org/0000-0002-9145-6059

Roberto A. Zucchi https://orcid.org/0000-0001-9861-7460

### Data availability

All of the data that support the findings of this study are available in the main text.
